# Complex role of miR-130a-3p and miR-148a-3p balance on drug resistance and tumor biology in esophageal squamous cell carcinoma

**DOI:** 10.1038/s41598-018-35799-1

**Published:** 2018-12-03

**Authors:** A. K. Eichelmann, C. Matuszcak, K. Lindner, J. Haier, D. J. Hussey, R. Hummel

**Affiliations:** 1 Department of General and Visceral Surgery, University Hospital Münster, Albert-Schweitzer-Campus 1, W1, 48149 Münster, Germany; 2 University Cancer Center Hamburg, Department of Pneumology, Martinistrasse 52, 20246 Hamburg, Germany; 3 Schön Klinik Hamburg Eilbek, Dehnhaide 120, 22081 Hamburg, Germany; 4 Nordakademie, Koellner Chaussee 11, 25337 Elmshorn, Germany; 5 Discipline of Surgery, College of Medicine and Public Health, Flinders University, Adelaide, South Australia Australia; 6 Department of Surgery, University Hospital of Schleswig-Holstein, Campus Lübeck, Lübeck, Germany

## Abstract

miRNAs play a crucial role in cancer development and progression. However, results on the impact of miRNAs on drug sensitivity and tumor biology vary, and most studies to date focussed on either increasing or decreasing miRNA expression levels. Therefore, the current study investigated the role of different expression levels of miR-130a-3p and miR-148a-3p on drug resistance and tumor biology in four esophageal squamous cell carcinoma cell lines. Interestingly, up- and downregulation of both miRNAs significantly increased sensitivity towards chemotherapy. MiRNA modulation also reduced adherence and migration potential, and increased apoptosis rates. Target analyses showed that up- and downregulation of both miRNAs activated the apoptotic p53-pathway via increased expression of either BAX (miR-148a-3p) or Caspase 9 (miR-130a-3p). miR-148a-3p downregulation seemed to mediate its effects primarily via regulation of Bim rather than Bcl-2 levels, whereas we found the opposite scenario following miR-148a-3p upregulation. A similar effect was observed for miR-130a-3p regulating Bcl-2 and XIAP. Our data provide the first evidence that miRNA modulation in both directions may lead to similar effects on chemotherapy response and tumor biology in esophageal squamous cell carcinoma. Most interestingly, up- and downregulation seem to mediate their effects via modulating the balance of several validated or predicted targets.

## Introduction

Esophageal cancer (EC) is characterised by poor 5-year survival rates of 15–34% due to, among other reasons, the development of chemotherapy resistance^1^. Over the past decade, several studies have demonstrated the importance of microRNAs (miRNAs) in cancer biology and their impact on response to chemotherapy in this tumor type^2,3^. Hence, miRNAs are potential diagnostic and therapeutic tools in the battle against esophageal cancer. miRNAs are short (19–24 nucleotides), single-strand, non-coding RNA sequences that have gained significant attention as potent regulators of gene expression in recent years^4^. They control numerous fundamental cellular processes by silencing target gene expression via posttranscriptional inhibition of translation through miRNA-mRNA interaction^5–7^. Therefore, it is not surprising that they have been identified as modulators of key cellular pathways associated with cancer-relevant processes such as proliferation, apoptosis, cell cycle control, differentiation, migration and metabolism^8–10^. Based on these promising data, modulation of miRNA levels might possibly open a new era of targeted molecular based therapy in cancer treatment. In support of this, multiple studies in recent years have shown that modulation of miRNAs impacts on cellular behaviour and response to chemotherapy in different tumor types^2,11,12^.

In our own most recent work, we demonstrated that several miRNAs including miR-130a-3p and miR-148a-3p impact on response to chemotherapy and biological behaviour in esophageal squamous cell carcinoma^2,3,13^. In detail, we showed that miR-130a-3p sensitized ESCC cells towards Cisplatin/5-FU in 100%/83% of tested cell lines, and miR-148a-3p in 83%/33%. Most interestingly, however, we observed that simultaneous manipulation of the expression levels of miR-130a-3p and miR-148a-3p exhibited additive sensitizing effects towards Cisplatin in 75% of cells^13^. A review of the current literature confirmed that these two miRNAs seem to play a crucial role in cancer biology as well as in response to chemotherapy. However, somewhat surprisingly, we found quite contradictory published results for both miRNAs on their actual role in cancer. For example, miR-130a was described as an oncogene or tumor suppressor gene in gastric^14,15^ or prostate^16^ cancer, and expression was either down-^17–19^ or upregulated^11,20^ in different tumor types. Further, miR-130a upregulation was shown to either increase^18^ or decrease^21^ sensitivity towards chemotherapy. Similar results were found for miR-148a which was described as an oncogene^22^ or tumour suppressor^23–26^. And again, with regards to biological behaviour in various cancer types, inconsistent results were reported with inhibition^23,26–30^ or promotion of aggressiveness^31,32^. In general, miRNAs, their function and their control of gene expression are believed to be tissue specific. This might, at least in part, explain these contradictory results in different tumor types^33^. However, for both miRNAs conflicting results have been described even for the same tumour type, such as gastric cancer^14,29,31,34^.

Based on these incongruous findings in the literature, and the fact that most studies focussed on either increasing or decreasing miRNA expression levels in tumors but did not investigate effects of alteration of miRNA expression levels in both directions, we selected these two miRNAs for a thorough investigation of their role in resistance and tumor biology in esophageal squamous cell carcinoma (ESCC). We modulated the expression of both miRNAs via transfection of mimics and inhibitors for each miRNAs in different ESCC cell lines. We then conducted analyses on chemotherapy response and biological behaviour, followed by a detailed analysis of targets and targeted pathways.

## Results

### Confirmation of successful transfection

Success of transient transfection using miRNA mimics and inhibitors was investigated using qRT-PCR. Transfection with miR-130a-3p and miR-148a-3p mimics resulted in increased expression of both miRNAs compared to scramble transfected controls (miR-130a-3p: 521–2030 ± 133-fold; miR-148a-3p: 1462–1530 ± 246-fold), and transfection with miR-130a-3p and miR-148a-3p inhibitors resulted in decreased expression of both miRNAs compared to scramble transfected controls (miR-130a-3p: -4–3 ± 0.5-fold; miR-148a-3p: -2–1 ± 0.09-fold; see Supplementary Fig. S2).

### Both, up- and downregulation of miR-130a-3p and miR-148a-3p increased sensitivity towards chemotherapy in ESCC

We then investigated the effect of up- and downregulation of miR-130a-3p and miR-148a-3p on response to chemotherapy treatment with Cisplatin and 5-FU in ESCC cell lines. We used four different ESCC cell lines in an attempt to mimic, at least in part, the inter-individual variability between cancer patients, and establish a more robust pre-clinical model for determining the relevance of our results.

**Figure 1:**
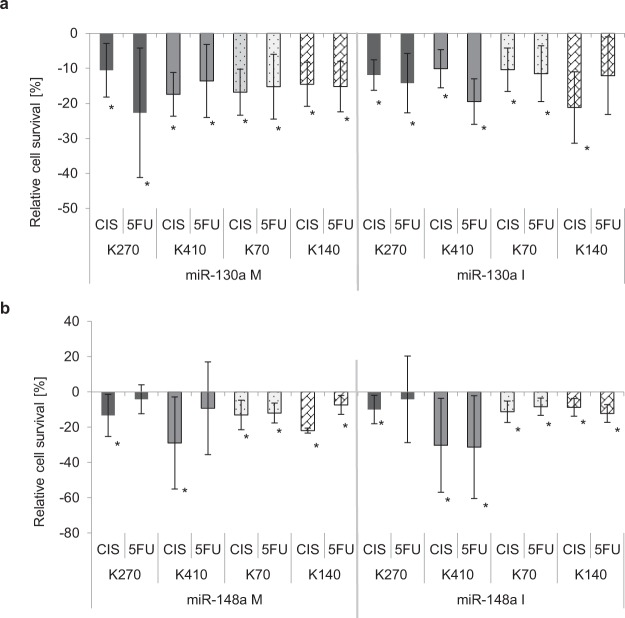
Chemotherapy response in esophageal cancer cell lines is affected by (**a**) miR-130a-3p and (**b**) miR-148a-3p. Response to chemotherapy after up- and downregulation of miRNA expression levels in four ESCC cell lines for miR-130a-3p (**a**) and miR-148a-3p (**b**). Cells were treated with chemotherapeutic agents at the respective LD50 dose 24 h after transfection with either mimic or inhibitor of miR-130a-3p or miR-148a-3p, and relative cell survival in relation to scramble-transfected control cells was measured 72 h after treatment with Cisplatin (Cis) or 5FU. At least six experiments were repeated independently. Data (student t-test) are presented as means ± standard deviation. *p < 0.05; control = 0%; M: Mimic; I: Inhibitor.

With this approach, we found that upregulation of miR-130a-3p significantly increased sensitivity towards both chemotherapeutic agents in all four ESCC cell lines by about 11–23% (p ≤ 0.03). Similarly, miR-148a-3p upregulation resulted in significantly increased sensitivity towards Cisplatin by about 13–29% (p ≤ 0.03) in all cell lines, and towards 5-FU by about 7–12% (p ≤ 0.03) in two of four tested cell lines. Most interestingly, however, miR-130a-3p downregulation also significantly increased sensitivity towards chemotherapy by about 10–21% (p ≤ 0.02) for Cisplatin in all cell lines and 12–20% (p ≤ 0.03) for 5-FU in 3 out of 4 cell lines. Similarly, miR-148a-3p downregulation significantly improved response towards Cisplatin in all cell lines, and towards 5-FU in 3 out of 4 cell lines (9–30% for Cisplatin, p ≤ 0.02 and 8–31% for 5-FU, p ≤ 0.04, Fig. 1).

### Both, up- and downregulation of miR-148a-3p inhibited adhesion and migration, and induced apoptosis

Next, we aimed to investigate if miRNA modulation affects other aspects of aggressive tumor biology. In detail, we analysed the effect of miR-148a-3p modulation on adhesion, migration and apoptosis. Again, we used two different cell lines derived from different patients.

**Figure 2:**
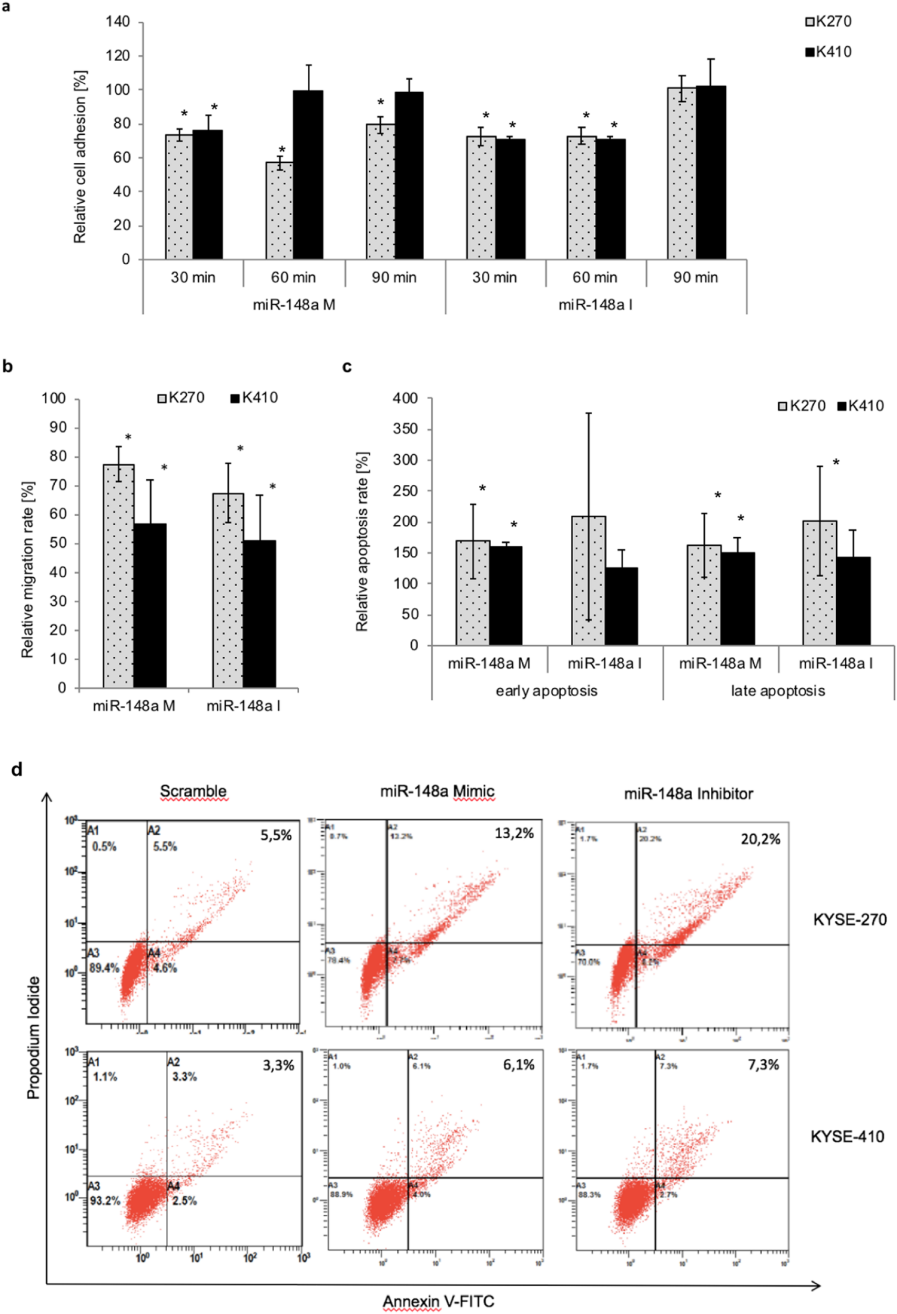
Effect of miR-148a-3p expression on biological behaviour in ESCC cell lines: adhesion, migration and apoptosis. Effect of miRNA modulation on adhesion (**a**) migration (**b**) and apoptosis (**c**,**d**) in ESCC cell lines KYSE-270 and KYSE-410. Relative migration and adhesion ratio compared to control was measured by crystal violet staining 48 h after transfection. Adhesion assays were performed five times; migration assays six times. Relative early and late apoptosis rate compared to control was measured by 7AAD/Annexin-FITC staining 48 h after transfection (**d**). Apoptosis experiments were performed in six independent replicates. Data (student t-test) are presented as means ± standard deviation. *p < 0.05; M: Mimic; I: Inhibitor; control = 100%.

Adhesion assays showed that the ability to adhere was significantly reduced by about 21–43% following miR-148a-3p upregulation in KYSE-270 cells after 30 to 90 minutes (p ≤ 0.0006) and by about 24% in KYSE-410 cells after 30 minutes (p = 0.01). Most interestingly, downregulation of miR-148a-3p also resulted in a significantly decreased adhesion (27–29%) after 30–60 minutes in both cell lines (p ≤ 0.004; Fig. 2a).

Comparable findings were observed for migration. Both, up- and downregulation of miR-148a-3p resulted in a significantly decreased ability to migrate in both cell lines by about 23–49% (p ≤ 0.001; Fig. 2b).

In addition to migration and adhesion assays, we found increased apoptosis rates following up- respectively downregulation of miR-148a-3p, especially for late apoptosis. Upregulation of miR-148a-3p resulted in increased apoptosis rates in both cell lines (50–62%, p < 0.03). Interestingly, downregulation of miR-148a-3p also increased apoptosis rates in KYSE-270 (102%, p = 0.04). Enhanced apoptosis levels were also observed for KYSE-410 (43%); however, data barely failed to reach statistical significance (p = 0.058).

### miR-130a-3p and miR-148a-3p regulate p53-dependent apoptosis-pathway

Finally, we aimed to identify modes of action of both miRNAs that are consistent with the observed effects on chemotherapy response and tumor biology. We previously demonstrated that miR-130a-3p and miR-148a-3p both target Bcl-2 directly, and both affect expression of XIAP (miR-130a-3p) and Bim (miR-148a-3p) in a direction consistent with negative posttranscriptional control^13^. Therefore, we analysed the effects of expression of both of these miRNAs on these molecules, and further downstream molecules in the p53-dependent apoptosis-pathway via Western Blot techniques.

**Figure 3:**
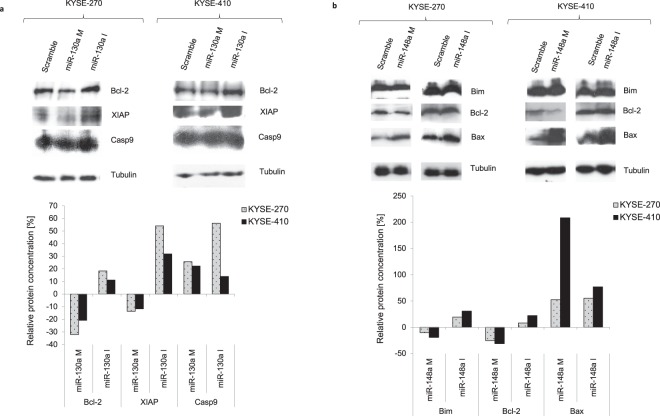
Western Blot analysis of potential targets of miR-130a-3p (**a**) and miR-148a-3p (**b**). Effect of miRNA manipulation on protein levels of direct and putative targets in ESCC cell lines. Relative protein levels compared to controls were measured in western blot analysis using antibodies 48 h after transfection. Proteins are listed according to their size. For reasons of clarity, the presented blots in this figure were cropped from different gels (represented by the white space). Full-length gels and blots can be found in the Supplementary Information File S3. Three independent Western experiments were performed. Bcl-2: B-cell lymphoma 2; XIAP: X-linked inhibitor of apoptosis; Casp9: caspase 9; M: Mimic; I: Inhibitor.

Following upregulation of miR-130a-3p, we found protein levels of anti-apoptotic Bcl-2 and pro-apoptotic XIAP to be decreased, as expected. Downregulation of miR-130a-3p resulted accordingly in the opposite effect, with increased protein levels of both Bcl-2 and XIAP. We noticed that the effect of miR-130a-3p upregulation on suppression of protein levels was more pronounced for Bcl-2 compared to XIAP, whereas the inhibition of miR-130a-3p resulted in a more pronounced increase of protein levels of XIAP compared to Bcl-2. Caspase-9 was analysed as a further downstream target in the activation of the p53-dependent apoptosis-pathway. We found that both, up- and downregulation of miR-130a-3p resulted in increased protein levels of Caspase-9 (Fig. 3a).

As expected, upregulation of miR-148a-3p led to decreased expression of pro-apoptotic Bim and anti-apoptotic Bcl-2 in both cell lines tested. Accordingly, downregulation of miR-148a-3p resulted in an increase of Bim and Bcl-2 expression levels. We found, similar to the above described pattern for miR-130a-3p, that the impact of miR-148a-3p upregulation on suppression of protein expression was more pronounced for Bcl-2 compared to Bim, whereas miR-148a-3p downregulation led to more pronounced upregulation of Bim compared to Bcl-2. The expression of Bax (which is not a potential target of miR-148a-3p but a downstream target in the p53-dependent apoptosis-pathway) was increased after both, up- and downregulation of miR-148a-3p (Fig. 3b).

**Figure 4:**
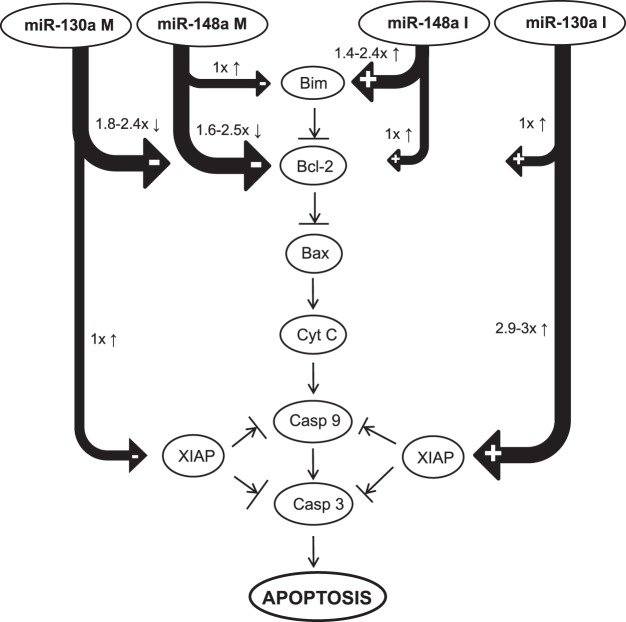
Analysis of p53-pathway in relation to miRNA expression and potential targets. Effect of miRNA manipulation on potential targets for miR-130a-3p and miR-148a-3p and their effect in p53-dependent apoptosis pathway.

Based on the observation of a different impact of miRNA up- or downregulation on different targets, we compared the ratios of XIAP versus Bcl-2 expression after miR-130a-3p modulation, and of Bim versus Bcl-2 expression after miR-148a-3p modulation. We found that miR-130a-3p inhibition seems to primarily mediate its effects via regulation of XIAP rather than Bcl-2 levels (XIAP to Bcl-2 ratio = 2.9 up to 3 depending on the cell line), and upregulation of miR-130a-3p seems to primarily mediate its effects via regulation of Bcl-2 rather than XIAP levels (Bcl-2 to XIAP ratio = 1.8 up to 2.4; see Fig. 4). A similar picture was found for miR-148a-3p, where miR-148a-3p downregulation seems to primarily mediate its effects via regulation of Bim rather than Bcl-2 levels (Bim to Bcl-2 ratio = 1.4 up to 2.4 depending on the cell line), whereas miR-148a-3p upregulation seems to primarily mediate its effects via regulation of Bcl-2 rather than Bim levels (Bcl-2 to Bim ratio = 1.6 up to 2.5; see Fig. 4).

## Discussion

In recent years, research has focussed on miRNAs and their role in cell regulation, differentiation, apoptosis, or their potential to modulate key cellular pathways and tumor development. This has led to an improved understanding of many of the fundamental processes and molecular mechanisms of cancer biology. Yet, our knowledge about the exact mechanisms mediating the effects of miRNAs on tumor biology is still limited. Studies from different groups on the same miRNA show contradictory results. Moreover, the majority of studies have mainly analysed the effect of either up- or downregulation of miRNAs levels in different tumor types and have not thoroughly investigated effects of alteration of miRNA expression levels in both directions. Recently, we introduced the concept that modified miRNA expression in both directions can interfere with the available degree of regulatory freedom of cellular functional complexes and thus change their functional readout^35^. Therefore, the current study aimed to investigate the impact of up- and downregulation of miR-130a-3p and miR-148a-3p on cellular behaviour and drug response in ESCC.

We observed that both, up- and downregulation of miR-130a-3p increased sensitivity to chemotherapy drugs in ESCC. We also observed an increase in sensitivity to these drugs upon both up- and downregulation of miR-148a-3p. We found that both up- and downregulation of miR-148a-3p inhibited adhesion and migration, and induced apoptosis. Taken together, our data showed that manipulating the expression levels of miR-130a-3p and miR-148a-3p in both directions led to similar effects on chemotherapy response, and modulation of miR-148a-3p levels in both directions affected the metastatic potential of cell lines in the same way. The target analyses showed that both miRNAs interact with the p53-dependent apoptosis-pathway. Interestingly, both, up- and downregulation of miR-130a-3p, and miR-148a-3p activated the p53-dependent apoptosis-pathway. Regardless of whether the miRNA of interest was up- or downregulated, the final steps of the pathway regulation were similar, namely activation of downstream targets Caspase-9 (miR-130a-3p) and Bax (miR-148a-3p). However, up- and downregulation of both miRNAs seemed to result in a shift in the ratios of target gene expression of different direct and potential targets.

The two miRNAs chosen for this study have been reported to play an important role in cancer biology and response to chemotherapy. However, data about their expression in esophageal cancer patients is still limited. miR-148a expression was reported to be downregulated in esophageal cancer^36^, as it has been reported to be in other tumor types^11,16,34,37,38^. miR-130a expression was found to be upregulated in esophageal cancer^39^, but its baseline expression differs between other malignancies^34,40^. Further, contradictory results between various studies on miR-130a and miR-148a, implicating either tumor suppressor^16,25^ or oncogene function^14,22^ in different tumor types, complicate interpretation of the limited data available so far. Interestingly, these conflicting results haven even been described for the same tumor type such as gastric cancer^14,31,34,41^. Finally, data on the direct influence of both miRNAs on chemotherapy resistance are very rare for esophageal cancer at this stage. In our own previous work, we demonstrated an increase in sensitivity to Cisplatin following down-regulation of miR-130a-3p or up-regulation of miR-148a-3p in esophageal squamous cell carcinoma^2,13^. Furthermore, our data showed a synergistic effect of both miRNAs on drug resistance^13^. Evidence for the influence of both of these miRNAs has been published widely in other tumor types. miR-130a influences response to chemotherapy in various tumor types such as ovarian cancer^11^, breast cancer^42^ and hepatocellular carcinoma^21^, and miR-148a was attributed a central role in drug response in renal^24^ and prostate cancer^43^. However, the literature reports partly contradictory results in terms of basal miR-130a expression and intrinsic chemoresistance of various cancer cell lines. While miR-130a expression was found to be down-regulated in resistant ovarian^17,18^, lung^19^, prostate^44^, hepatocellular^40^ and head and neck squamous cell carcinoma cell lines^45^, the same miRNA was overexpressed in resistant ovarian cancer cells^11,20^. Consequently, modulation of miR-130a levels by upregulation can either sensitise cells to chemotherapeutic agents^18^ or contribute to drug resistance^21^.

An explanation for these apparently contradictory observations might lie in the fact that most prior studies focussed on analysing the effects on drug response following either increasing or decreasing miRNA expression levels. Of the small number of authors who did investigate the effects of alteration of miRNA expression levels in both directions, they often used different cell lines for their transfection studies (e.g. miR-148a up-regulation in lung carcinoma cell lines with endogenous low miR-148a expression (SPC-A-1sci, H1299, and A549) and miR-148a down-regulation in cell lines with endogenous high miR-148a expression (SPC-A-1, LC-1, H358)^26^. There are very few published studies in which modulation of expression of a single miRNA has been performed in both directions in the same cell line. However, in contrast to our findings, these studies mostly observed direction dependent effects, e.g. increase in apoptosis or chemosensitivity following modulation of the miRNA of interest in one direction, and decrease in apoptosis or chemosensitivity when expression of the miRNA was modified in the other direction. In ovarian cancer for example, miR-130a over-expression contributed to Cisplatin resistance, while down-regulation sensitised cells to Cisplatin^11,46^. These findings are in line with data from hepatocellular carcinoma^21^. Further, Wang *et al*. overexpressed miR-130a-3p in gastric cancer cell lines and observed an inhibition of migration, invasion and epithelial-mesenchymal transition *in vitro* and of tumorigenesis and lung metastasis *in vivo*. The exact opposite effects occurred, when miR-130a-3p was downregulated^34^. Authors, who analysed the effect of miR-148a modulation in opposite directions observed a similar phenomenon. In lung cancer for example, upregulation of miR-148a diminished the potential to proliferate and invade, as well as decreased apoptosis rates. Conversely, miR-148a inhibition accelerated migration and proliferation^47^. Yu *et al*. described the same phenomenon in gastric cancer cells^48^. However, it is worth emphasising that - again - findings from different studies investigating the same tumor type are contradictory. For example, Guo *et al*. described, in contrast to the findings mentioned above, that miR-148a inhibition leads to decreased proliferation rates of gastric cancer cells, and miR-148a over-expression leads to increased proliferation rates^31^. These findings are in line with the work from Murata in prostate cancer^32^. An increased ability to migrate and invade, as well as higher growth rates following miR-148a overexpression were also observed by Kim *et al*. in glioblastoma cell lines. Conversely, the opposite effects occurred when miR-148a was down-regulated^22^. Nevertheless, findings of all mentioned studies are in agreement insofar as all studies observed the exact opposite effect when expression levels of the miRNA of interest was altered in the opposite direction. This is in contrast to our results. However, considering the very large number of studies on miRNAs and their impact on metastatic potential and drug sensitivity, the number of studies investigating effects of alteration of miRNA expression levels in both directions is surprisingly low.

Our results revealed enhanced sensitivity towards chemotherapy as well as a decreased metastatic potential following up- and downregulation of miR-130a-3p and miR-148a-3p. To the best of our knowledge, this phenomenon has not previously been reported for miR-130a-3p nor miR-148a-3p, nor for any miRNA, in ESCC. However, in line with our findings, an identical scenario was described for miR-31 in pancreatic carcinoma. Laurila and colleagues up- and downregulated miR-31 expression levels in several pancreatic cancer cells. Surprisingly, alteration of miRNA levels in both directions resulted in similar phenotypic effects. For example, knockdown as well as enhanced miR-31 expression in AsPC-1 cells decreased the ability to migrate (miR-31 inhibition: −33%, miR-31 upregulation: −61%) or invade (miR-31 inhibition: −27%, miR-31 upregulation: −74%). The authors noted that even though the basal miR-31 expression in AsPC-1 is known to be high, up-regulation still led to a significant shift in cellular behaviour. From their data, the authors suggested that the amount of miR-31 is critical for the phenotypic effects^12^. Our current work in ESCC supports these findings, and highlights the importance of balance of intracellular miRNA expression, which plays a crucial role in cell behaviour.

In addition to functional analyses, the present study included a detailed pathway analysis following up- and downregulation of both miRNAs. Quite unexpectedly, both up- and downregulation of the two miRNAs resulted in activation of the p53-dependent apoptosis pathway. While downregulation of miR-130a-3p resulted in increased Bcl-2 and XIAP levels, and upregulation in the opposite effect, both up- and downregulation of miR-130a-3p increased levels of Caspase. The same effect was observed for miR-148a-3p. While up- and downregulation resulted in opposite regulation of Bim and Bcl-2, expression of Bax was increased following up- and downregulation (Fig. 3). Most interestingly, however, we observed that up- or downregulation resulted in a shift in the ratios of expression of different direct and potential targets (Fig. 4). In this context, we acknowledge that our finding of increased XIAP expression especially after miR-130a-3p inhibition are somewhat unexpected, as XIAP is generally considered to inhibit apoptosis via inhibition of caspases. Moreover, we acknowledge that our current data do not prove XIAP and Bim to be direct targets. However, our data clearly suggest that up- or downregulation of both miRNAs affect expression of several anti- and pro-apoptotic targets in the p53-dependent apoptosis pathway, and the specific balance of all these targets seems to determine the effect of miRNA manipulation on tumor biology and drug sensitivity. Available data suggests that miR-130a-3p and miR-148a-3p both play an important role in the intrinsic apoptotic pathway, which is recognized as a key step in tumorigenesis. Our previous experiments in ESCC showed that miR-130a-3p and miR-148a-3p both target Bcl-2 directly, which is an important molecule for regulating the apoptotic pathway^13^. MiR-148a was also found to induce cell apoptosis by targeting Bcl-2 in colorectal and pancreatic cancer^23,30^. For example, ectopic expression of miR-148a induced apoptosis by suppression of Bcl-2 and activation of a caspase cascade in colorectal cancer cells^30^.

We acknowledge that the selected miRNAs might exert their effects not only via the targets and pathways analysed in this study. In our previous work, we reported that a number of miRNAs, including miR-148a and miR-130a, might regulate a variety of targets in different resistance-relevant pathways including p53-dependant pathway of apoptosis, DNA-methylation or histone modification^13^. Other authors have reported a number of additional resistance-, biology- or EMT-relevant (potential) targets and pathways of miR-148a and miR-130a such as ERBB3/AKT2/c-myc, ERBB3/AKT2/Snail, CCKBR, SMAD2, TGF-β/Smad, Myc respectively TBL1XR1, TNF-α, RAB5A or RUNX3^15,23,34,38,49–54^. These findings appear to be in line with the hypothesis that the availability and balance of miRNAs determine the degree of regulatory freedom^35^ and, therefore, modulate the sensitivity of the apoptotic switch. To the best of our knowledge, no other studies have been published that included a detailed pathway analysis following miRNA modulation in both directions, as we report here. Our results might bring into question the conclusions drawn in former studies that investigated effects of miRNA modulation in only one direction. On the basis of our results, we believe that the balance of miRNA levels plays an important role in the cellular behaviour of ESCC.

One of the main limitations of this study, that may affect the relevance of our findings, is the fact that we included ESCC cells only and performed our work in *in-vitro* studies only. However, since current literature focussing on the impact of intracellular miRNA balance in cancer is very limited and there is no data addressing this topic in ESCC, the data collected from this *in-vitro* work provides an essential basis for follow-up projects. Moreover, we tried to address the argument of limited transferability of *in-vitro* cell culture results into clinical settings by including four different cell lines originating from four different patients in order to attempt to mimic - at least in part - the inter-individual variability. A second limitation is the fact that we cannot finally prove at this stage why and how both up- and downregulation of same miRNA result in same effect. To fully answer this question and to provide final prove about the exact mechanisms of action, reproduction of our results by others is mandatory, and additional target genes and target pathways of these two miRNAs have to be further investigated. However, we present with this current work first evidence of this phenomenon in esophageal squamous cell carcinoma, and provide a basis for future research into this highly interesting observation. Finally, we acknowledge the known problem that mimic transfection into cells results in fairly high expression levels above those associated with physiological variation. It is established, and has been published, that overloading cells with miRNA mimics actually results in artefacts in expression^55^.

In summary, the current work suggests that the balance of miR-130a-3p and miR-148a-3p expression plays a crucial role in drug resistance and tumor behaviour in ESCC via regulation of the p53-dependent apoptosis pathway. Our results provide new insight into the mechanisms of action of miRNAs, and highlight the complexity of their role in epigenetic regulation of metastatic behaviour and apoptosis. Further studies are needed to verify our observations in other tumor types.

## Methods

### Cell culture

Human ESCC cell lines KYSE-70, KYSE-140, KYSE-270 and KYSE-410 (obtained from Deutsche Sammlung von Mikroorganismen und Zellkulturen (DSMZ; Braunschweig, Germany)) were used. All cell lines, except KYSE-270, were cultured using RPMI 1640 medium (Lonza, #BE12-918F), supplemented with 10% FCS (Biochrom, #S0615) and 5% L-glutamine (Lonza, #BE17-605E). KYSE-270 was cultured using 50% RPMI 1640 medium and 50% Hams medium (Biozol, #1-14F22-I) with 10% FCS and 5% L-glutamine. Cells were cultured using standard techniques and reagents as described previously^2,3,56–58^.

### Transient transfection

The four ESCC cell lines KYSE-70, KYSE-140, KYSE-270 and KYSE-410 were grown to 60–80% confluence, followed by a transfection with either mimics or inhibitors of miR-130a-3p or miR-148a-3p (Qiagen, #MSY0000243, #MSY0000425, #MIN0000243, #MIN0000425), or the negative scramble-control (Qiagen, #SI03650318) using Lipofectamine 2000 transfection agent (Life Technologies, #11668027). The slightly modified manufacturer’s protocol has been described previously^58^. See Supplementary Table S1 for cell numbers seeded in the 6-well plates.

### RNA extraction and qRT-PCR

48 h after transfection (=equivalent time point to treatment and characterization studies), transfection efficiency was measured through real-time quantitative PCR (miScript PCR system, Qiagen, #218161). For this purpose, cells were lysed using TRIzol reagent (Invitrogen Life Technologies) and total RNA was extracted using the miRNeasy Kit (Qiagen, #217004) according to the instructions of the manufacturer. RNA concentration was determined using UV spectrophotometry. MiScript assays were then performed as described previously^58^.

### Drug treatment

Transfected cells were treated 24 h post-transfection with Cisplatin (Teva GmbH) or 5-FU (Medac GmbH), which represent the standard chemotherapy treatment for esophageal cancer. For this purpose, the cells were treated with IC50 doses of each drug (see Supplement S1) for 72 h. Cell proliferation was then analysed by a MTT assay using Thiazolyl Blue Tetrazolonium Bromide (Sigma-Aldrich, #M2128-1G). The protocol for the drug treatment and the MTT assay has been described previously^58^. Results were verified in at least six independent experiments, and each experiment included four technical replicates.

### Adhesion and migration assays

KYSE-270 and KYSE-410 cell lines were used to analyse the effect of miR-148a-3p manipulation on adhesion and migration. Details for the adhesion assay can be found in our previous publication^58^. Results were verified in five independent experiments, and each experiment included four technical replicates.

For analysis of migration, cells (KYSE-270: 60,000; KYSE-410: 25,000) were plated onto an upper chamber of a 24-well Boyden chamber coated with type I collagen (2.5 µM) and fibronectin (50 nM) with an 8 µm pore polycarbonate membrane in medium without serum (Supplement S1). Medium containing 10% fetal bovine serum was filled in the lower chamber as chemoattractant. After 18 h, cells, which did not migrate through the pores, were removed using cotton swabs. After incubation in formalin and staining with crystal violet (15 minutes each), migrated cells were counted in 9 gridded high-power fields per membrane under an inverted microscope. The experiment was performed six times.

### Apoptosis analysis

Apoptosis assays through flow cytometry (Annexin V/ 7AAD; BioLegend, #640905) were performed after up- and downregulation of miR-148a-3p in KYSE-270 and KYSE-410 as described previously^58^. For flow cytometry, 20,000 events were collected (software for data acquisition: CXP software by Beckman Coulter) and cells were distinguished as follows: early apoptotic (Annexin V positive and 7AAD negative) and late apoptotic cells (Annexin V and 7AAD positive). Apoptosis assays included six independent replicates.

### Western blot

Our previous experiments that showed that Bcl-2 (B-cell lymphoma 2) is directly targeted by miR-130a-3p and miR-148a-3p. They also showed that expression of XIAP (X-linked inhibitor of apoptosis protein) is downregulated by miR-130a-3p, and that expression of Bim (Bcl-2-like protein 11) is downregulated by miR-148a-3p^13^. Therefore, we analysed the effect of miR-148a-3p expression on expression of these targets in combination with further downstream targets in the p53-dependent apoptosis-pathway (Bax and Caspase-9). For miR-130a-3p, we analysed Bcl-2 and XIAP and again Caspase-9 in the p53-dependent apoptosis-pathway. Cells were lysed 48 h after transfection with either mimic or inhibitor of miR-130a-3p or miR-148a-3p and processed according to a standard protocol as described previously^58^. Primary antibodies were purchased from BD Bioscience (anti-Bcl-2 #551097, anti-Bim #559685, anti-XIAP #610716), Santa Cruz Biotechnology (anti-Bax #sc-493), and Cell signaling (anti-Casp9 #9502). Three independent Western experiments were performed.

### Statistical analysis

The effect that modulating miRNA expression had on the response to chemotherapy was analysed as described previously^58^. Briefly, the mean corrected absorbance of the (scramble/mimic/inhibitor) transfected treated cells was normalized in a first step to the corresponding transfected but untreated controls. Secondly, the effect of mimic-/inhibitor-transfection on chemotherapy response was then presented as relative survival of mimic-/or inhibitor-transfected cells compared to scramble transfected controls (given in percent), which have been previously set to “0”. For Western blot experiments, we performed three independent experiments in each case. These three respective independent experiments showed in all cases comparable results. However, as there was a high inter-experimental variability of absolute results (relative protein concentration in %), we decided to present the results of one representative experiment and to point out, that results have been reproduced in another 2 independent experiments.

Student’s t-test for equal and unequal variances was used to compare between groups. We considered a p < 0.05 to be statistically significant. Statistical analyses were calculated by SPSS 21.0 (SPSS, Chicago, IL) and all data are expressed as means ± standard deviation.

## Electronic supplementary material

Cell numbers, cell line dependent IC50s and Western Blots

Dataset 1
