# Supplementary material for: Complex role of miR-130a-3p and miR-148a-3p balance on drug resistance and tumor biology in esophageal squamous cell carcinoma
Source: Sci Rep. 2018 Dec 3;8:17553. doi: 10.1038/s41598-018-35799-1 (PMC6277408; doi:10.1038/s41598-018-35799-1)
Supplement: Supplementary file 1 — Cell numbers, cell line dependent IC50s and Western Blots [file 41598_2018_35799_MOESM1_ESM.pdf]

Complex role of miR-130a-3p and miR-148a-3p balance on drug resistance and tumor biology in esophageal squamous cell carcinoma

Eichelmann AK, Matuszcak C, Lindner K,
Haier J, Hussey DJ, Hummel R

Supplement 1: Cell numbers and cell line dependent IC50 doses

Cell line	Cell number 96-well	Cell number 6-well	Cell number migration assay	IC50	
				Cis	5-FU
KYSE-70	8,000	100,000	/	10 µM	7 µM
KYSE-140	7,500	100,000	/	2 µM	20 µM
KYSE-270	3,000	125,000	60,000	3.5 µM	4.5 µM
KYSE-410	2,500	100,000	25,000	7 µM	20 µM

Supplement 2: Confirmation of successful transfection (after 48h) using qRT-PCR approaches. Both, up- and downregulation of the respective miRNA led to significant changes in miRNA expression. M: Mimic, I: Inhibitor; K410: KYSE-410, K270: KYSE-270; miR: miRNA

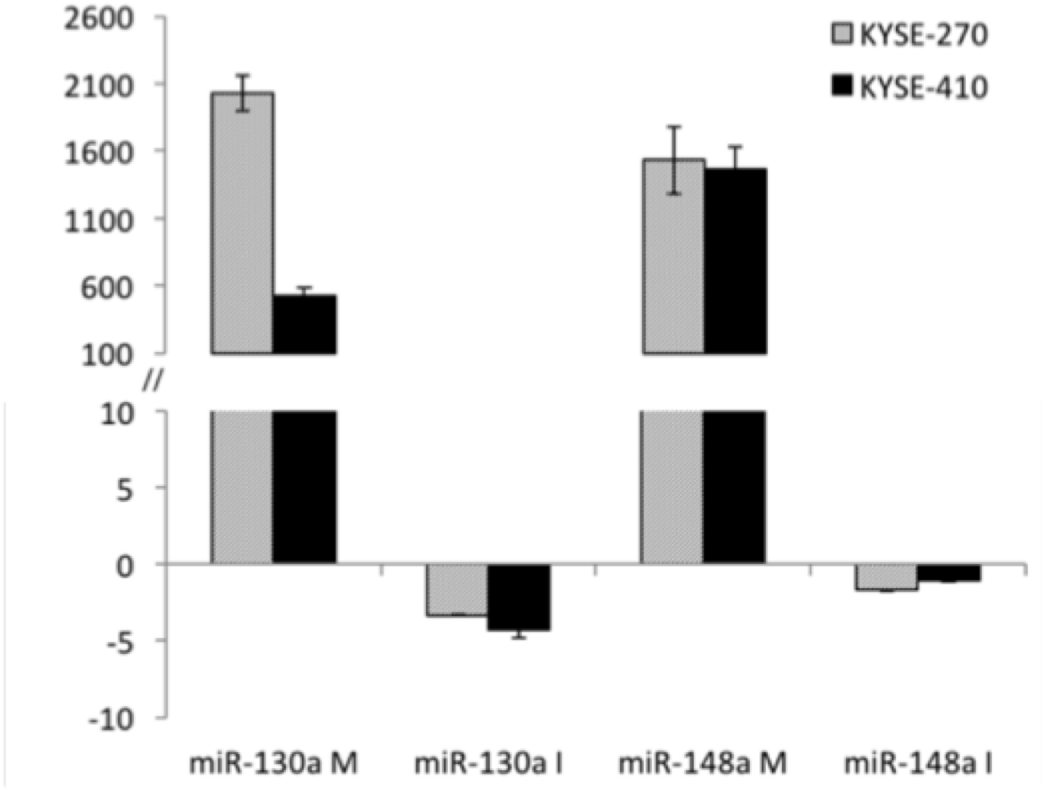

miR-130a
Bcl-2

Publication

anti-Bcl2 (miR-130/K270a)

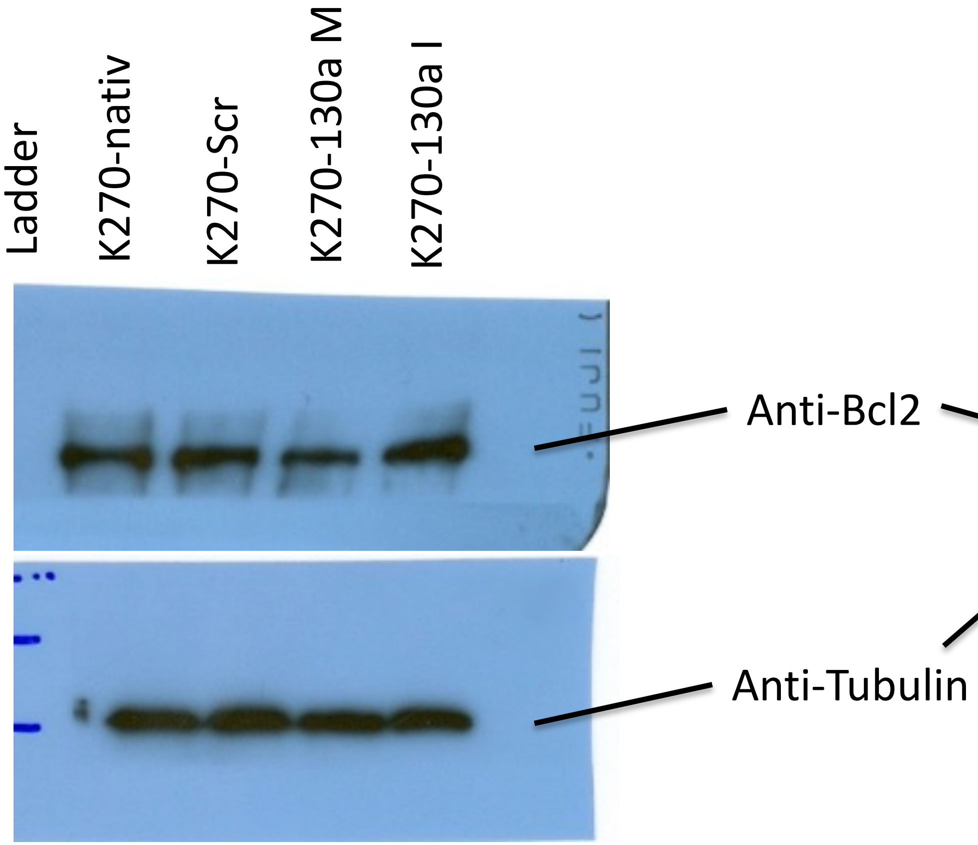

Brightness: +42%
Contrast: +35%

anti-Bcl2 (miR-130/K410a)

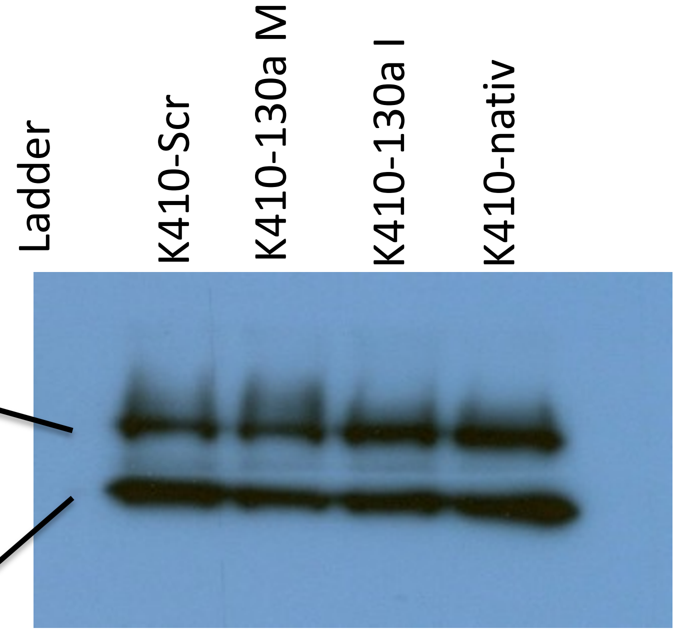

Brightness: +51%

Second experiment

anti-Bcl2 (miR-130)

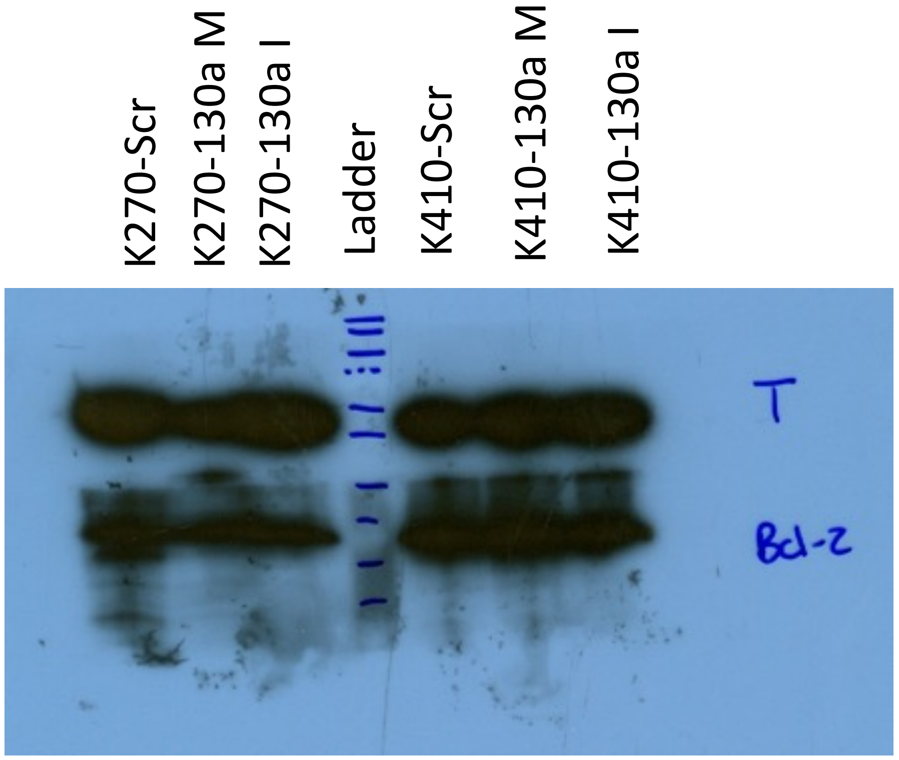

Third experiment

anti-Bcl2 (miR-130)

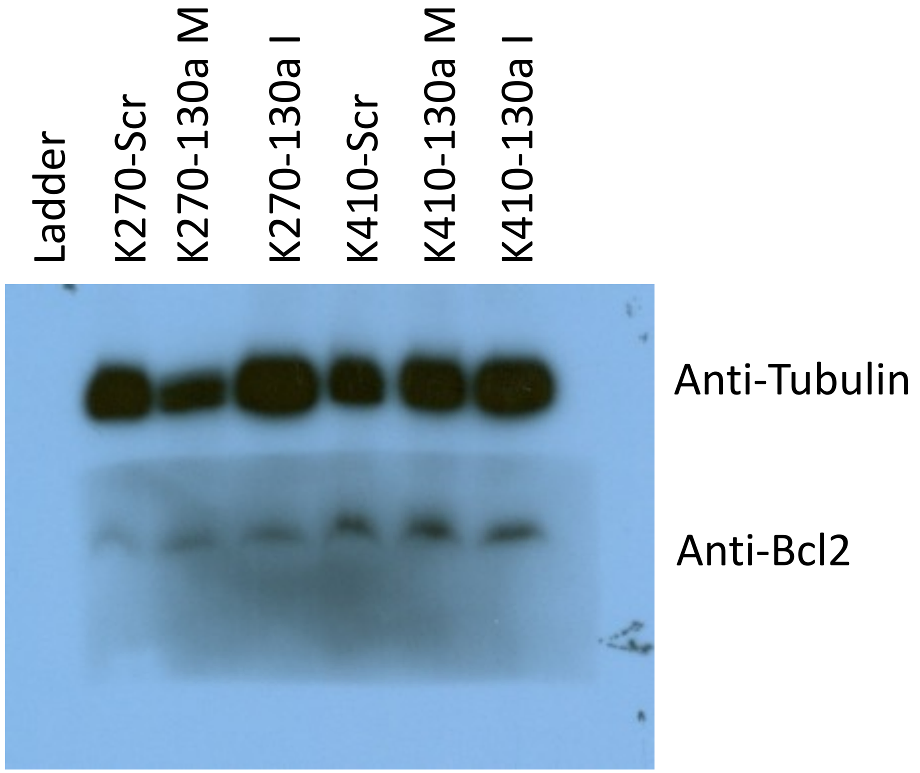

miR-130a
XIAP

Publication

anti-XIAP (miR-130a/K270)

Ladder
K270-Scr
K270-130a M
K270-130a I

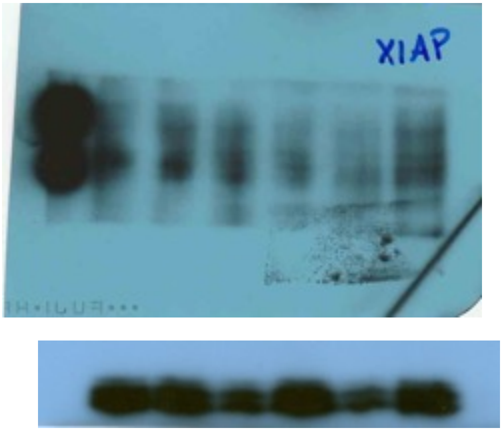

Anti-XIAP

Anti-Tubulin

Brightness: +18%
Contrast: +60%
Sharpening: +65%

anti-XIAP (miR-130a/K410)

Ladder
K410-Scr
K410-130a M
K410-130a I

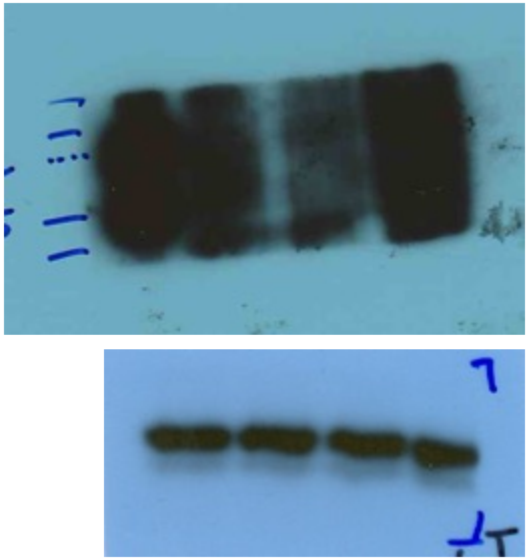

Brightness: +57%

Second experiment

anti-XIAP (miR-130a/K270)

anti-XIAP (miR-130a/K410)

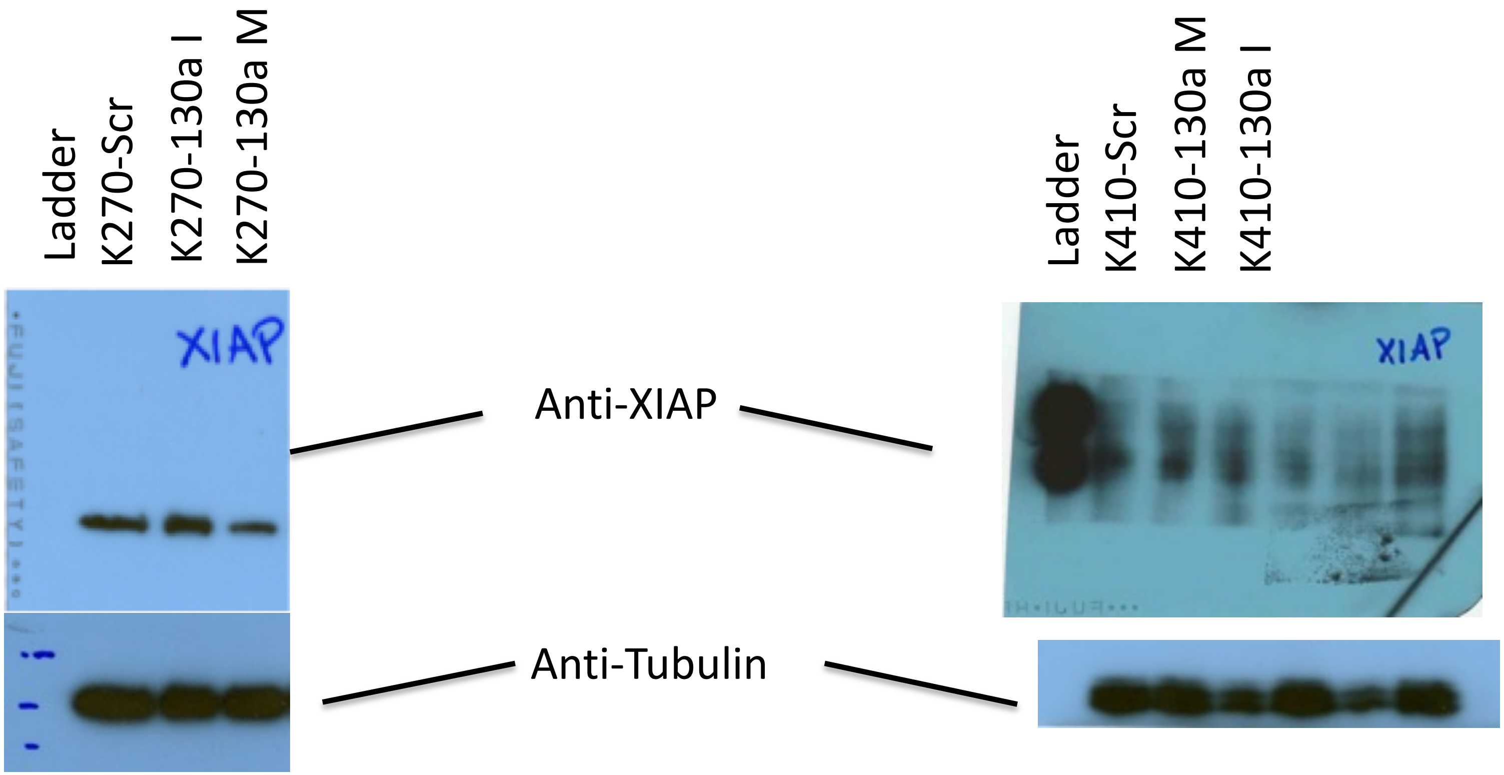

Third experiment

Anti-XIAP (miR-130)

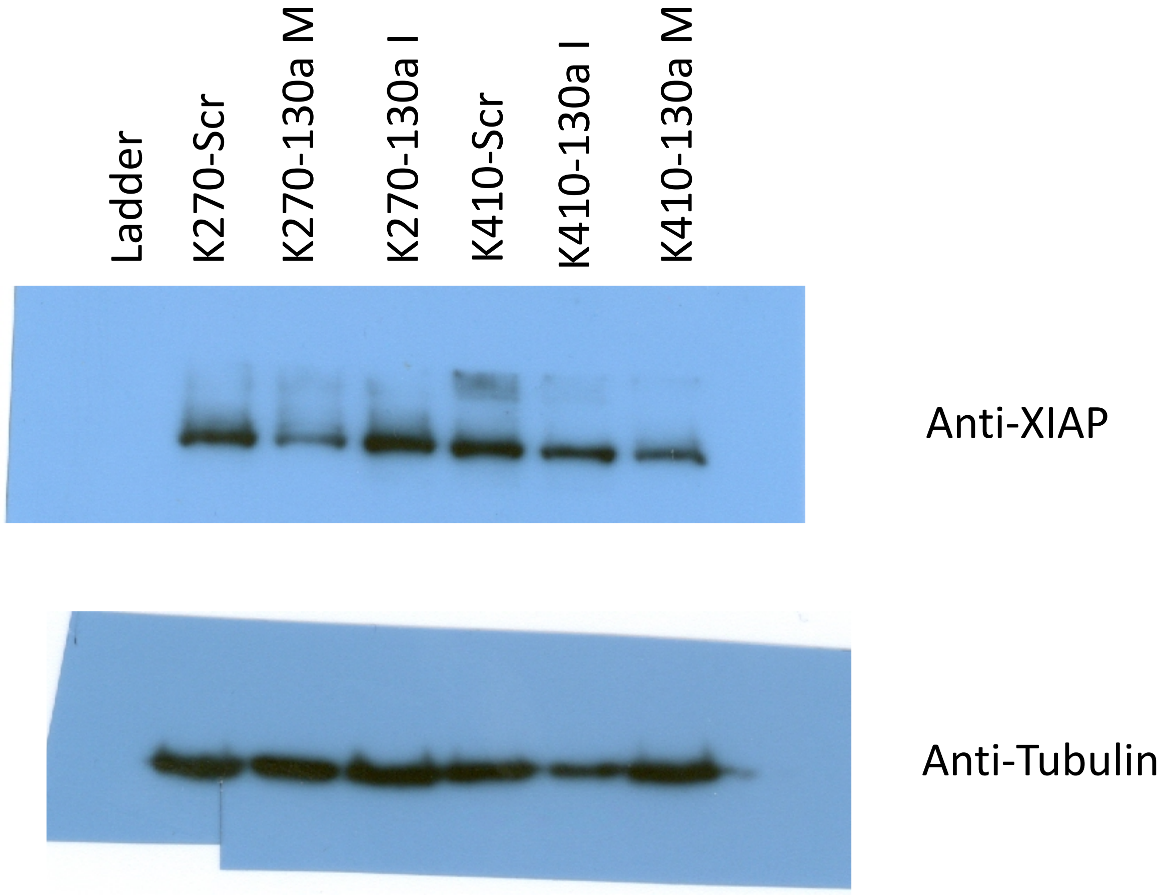

miR-130a
Casp9

Publication

anti-Casp9 (miR-130a/K270+K410)

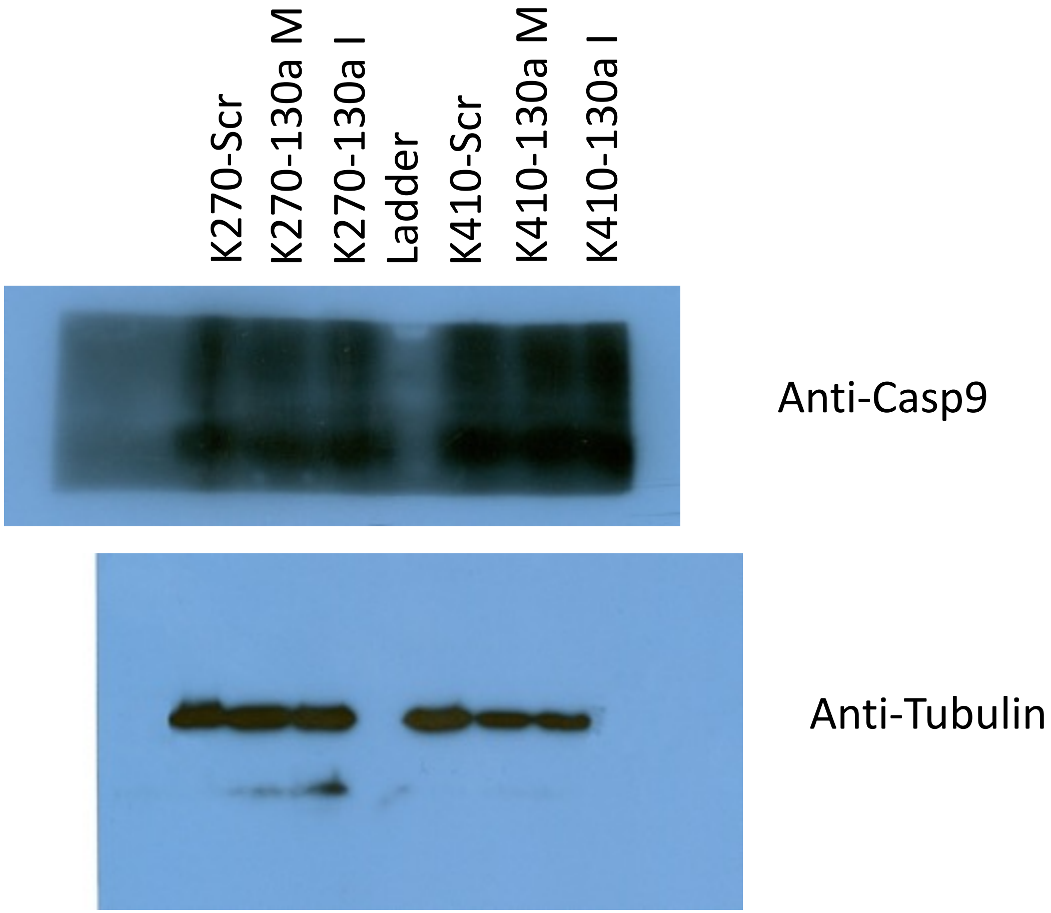

Brightness: +72%
Contrast: +66%
Sharpening: +44%

Second experiment

Anti-Casp9 (miR-130a/K270)   Anti-Casp9 (miR-130a/K410)

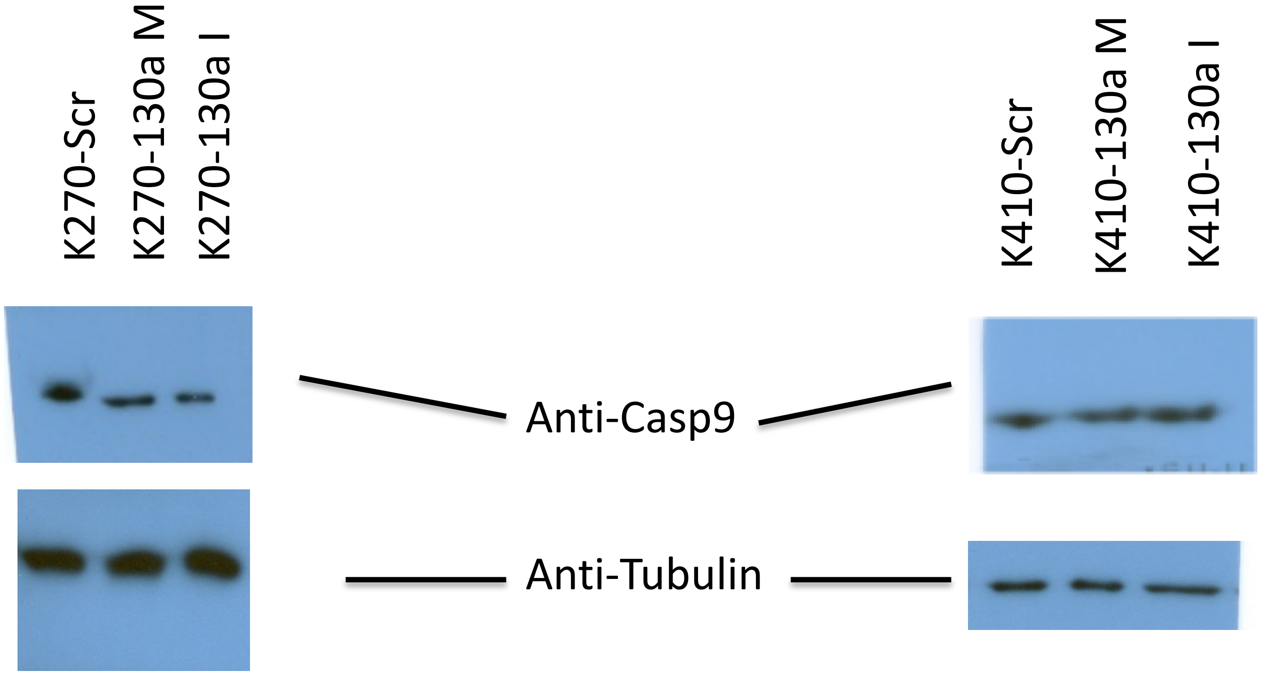

Third experiment

Anti-Casp9 (miR-130a/K270)

Anti-Casp9 (miR-130a/K410)

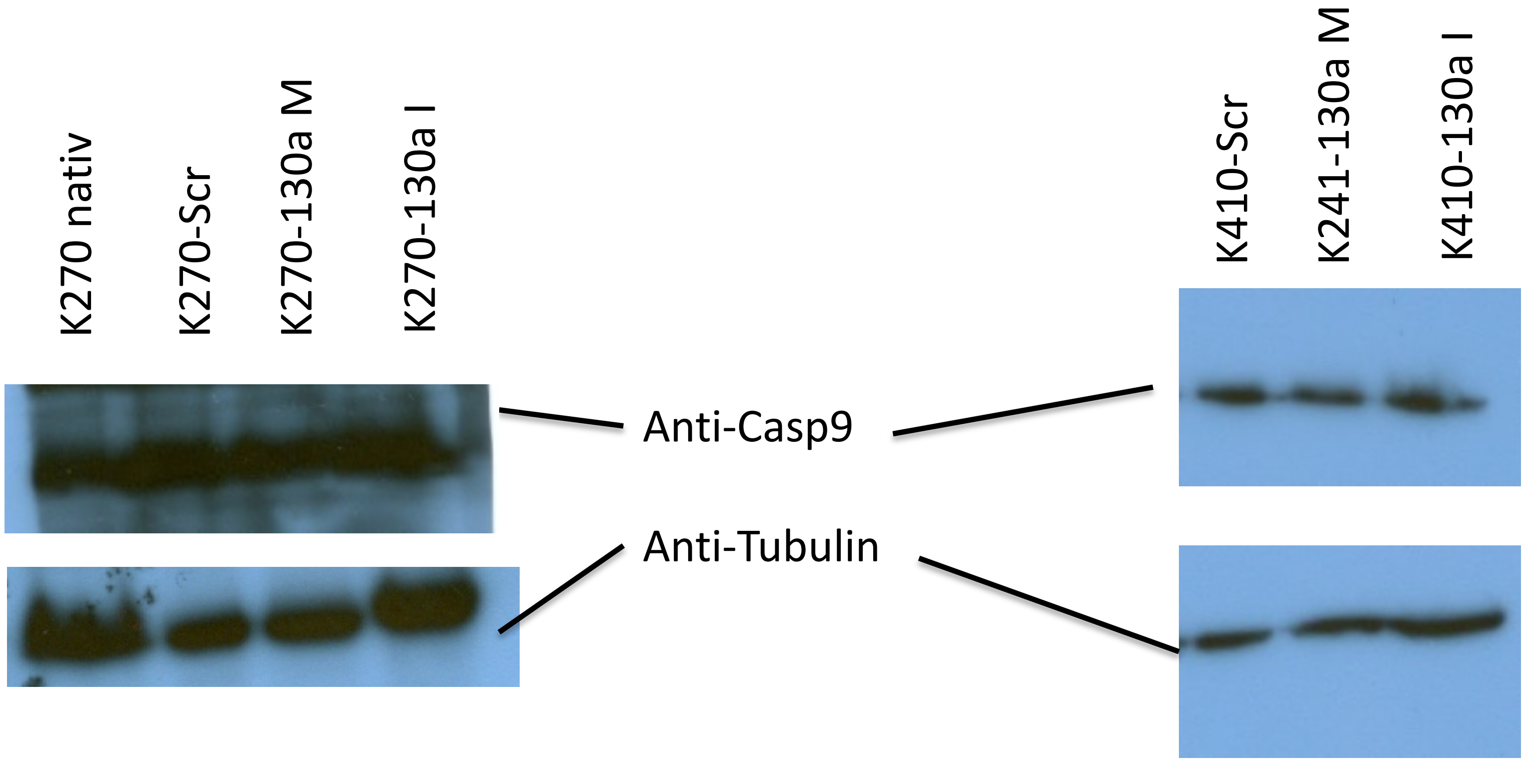

miR-148a
Bim

Publication

anti-Bim (miR-148a/K270+K410)

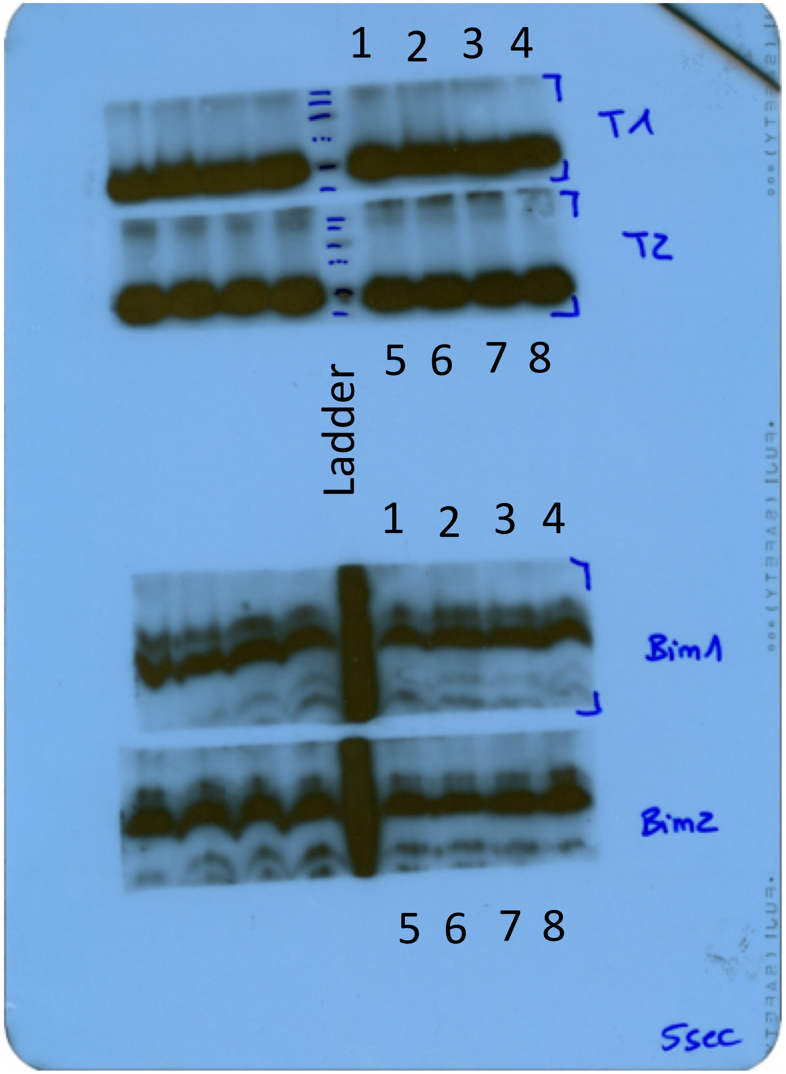

Anti-Tubulin

Anti-Bim

1 K410: Scr
2 K410: miR-148a Inhibitor
3 K270: Scr
4 K270: miR-148a Inhibitor
5 K270: Scr
6 K270: miR-148a Mimic
7 K410: Scr
8 K410: miR-148a Mimic

Brightness: +56%
Contrast: +48%

Second experiment

Anti-Bim (miR-148a)

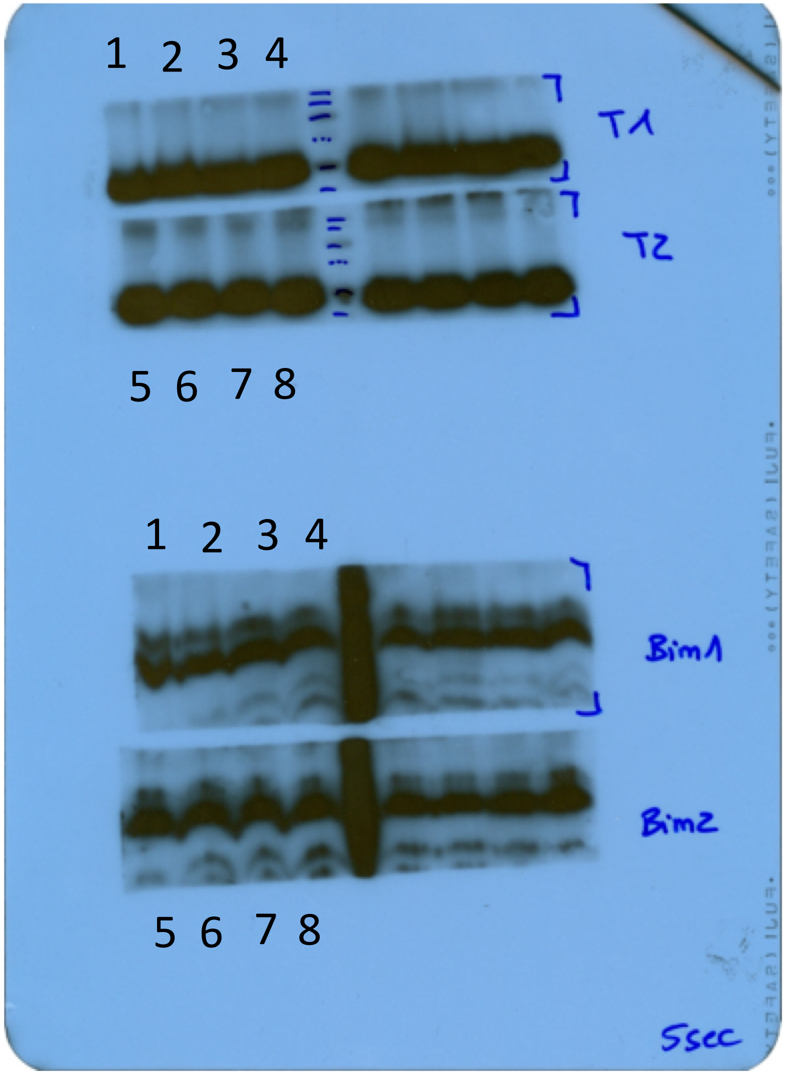

Anti-Tubulin

Anti-Bim

1 K410: Scr
2 K410: miR-148a Inhibitor
3 K270: Scr
4 K270: miR-148a Mimic
5 K270: Scr
6 K270: miR-148a Mimic
7 K410: Scr
8 K410: miR-148a Inhibitor

Third experiment

Anti-Bim (miR-148a)

1 K410: Scr
2 K410: miR-148a Inhibitor
3 K270: Scr
4 K270: miR-148a Inhibitor
5 K270: Scr
6 K270: miR-148a Mimic
7 K410: Scr
8 K410: miR-148a Mimic

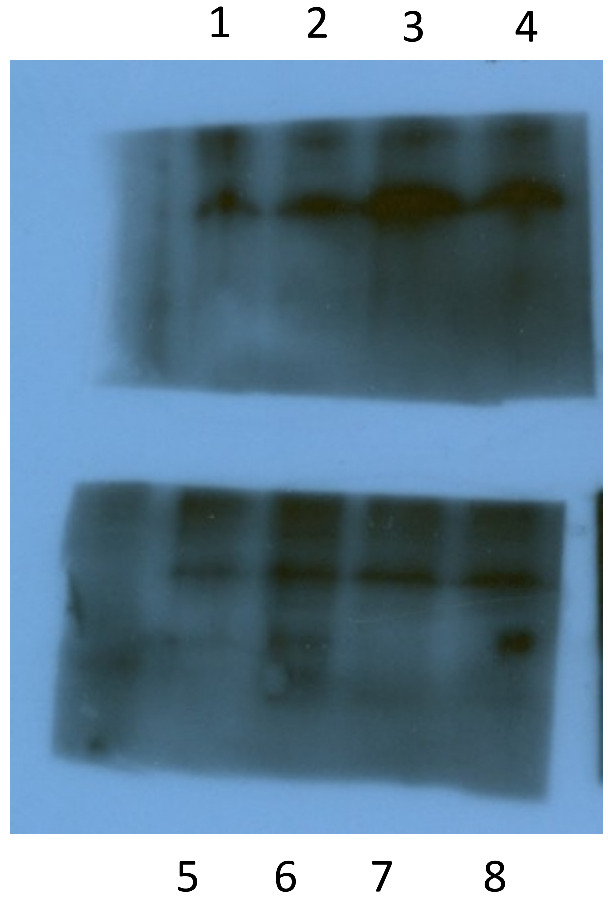

Anti-Bim

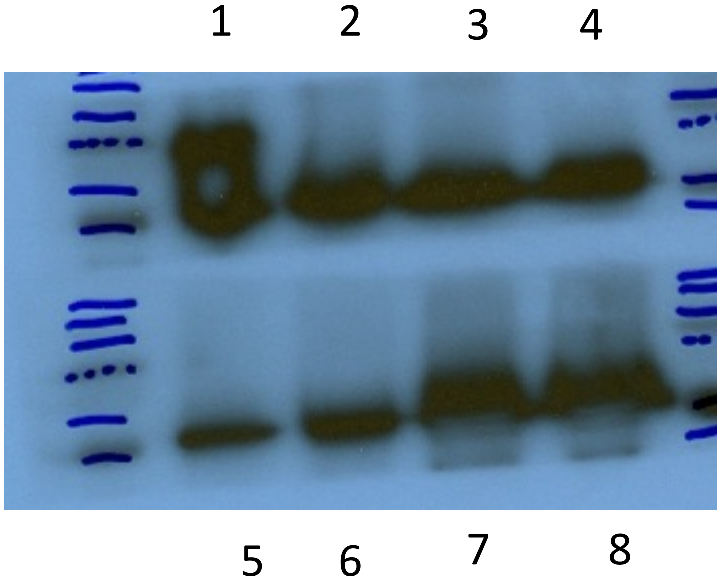

Anti-Tubulin

miR-148a
Bcl-2

Publication

anti-Bcl2 (miR-148a/K270)

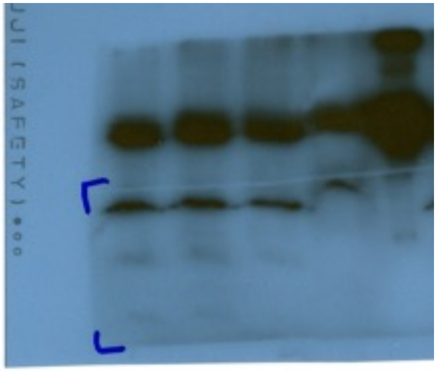

Anti-Tubulin

Anti-Bcl2

1 2 Ladder

1 K270: Scr
2 K270: miR-148a Mimic
3 K270: Scr
4 K270: miR-148a Inhibitor

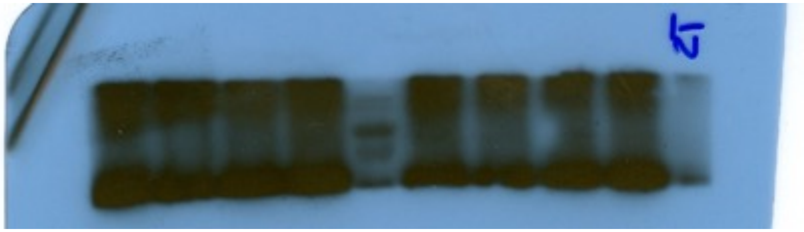

Anti-Tubulin

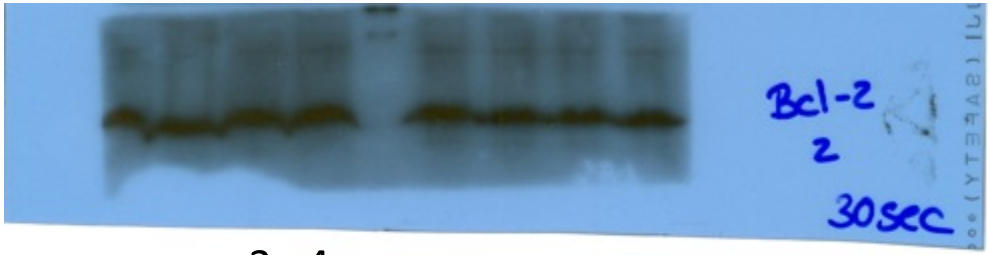

Anti-Bcl2

3 4

Brightness: +50%
Contrast: +40%

Publication

anti-Bcl2 (miR-148a/K410)

1 K410: Scr
2 K410: miR-148a Mimic
3 K410: Scr
4 K410: miR-148a Inhibitor

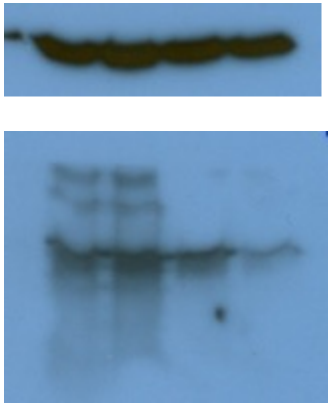

Anti-Tubulin

Anti-Bcl2

Ladder 1 2

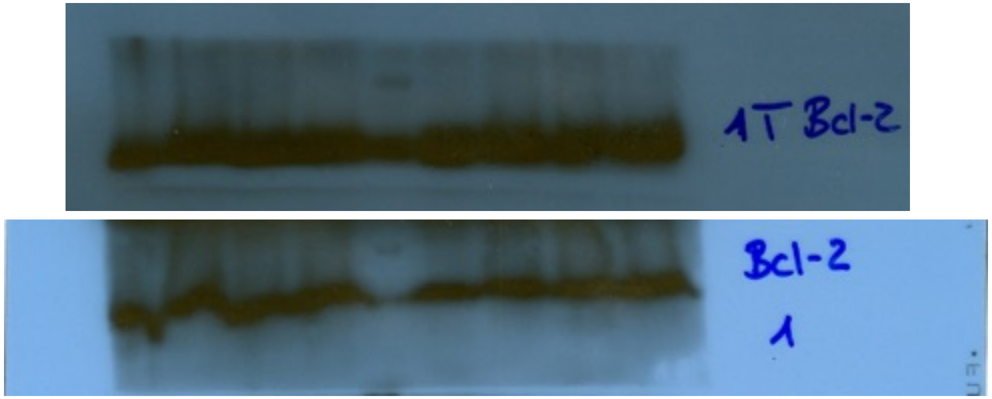

Ladder 3 4

Brightness: +50%
Contrast: +40%

Second and third experiment

Anti-Bcl-2 (miR-148a/K270)

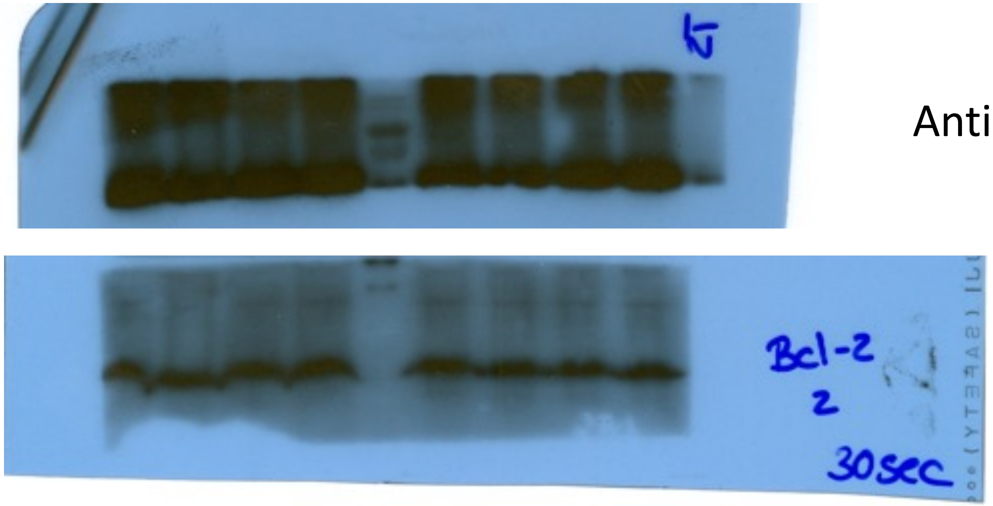

Anti-Tubulin

Anti-Bcl2

1 2 3 4 5 6

7 8

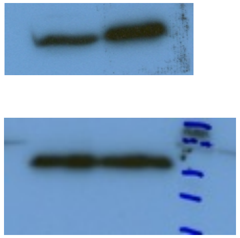

Anti-Bcl2

Anti-Tubulin

1 K270: Scr
2 K270: miR-148a Mimic
3 K270: Scr
4 K270: miR-148a Mimic
5 K270: Scr
6 K270: miR-148a Inhibitor
7 K270: Scr
8 K270: miR-148a Inhibitor

Second and third experiment

Anti-Bcl-2 (miR-148a/K410)

1 K410: miR-148a Inhibitor
2 K410: Scr
3 K410: Scr
4 K410: miR-148a Inhibitor
5 K410: Scr
6 K410: miR-148a Mimic
7 K410: Scr
8 K410: miR-148a Mimic

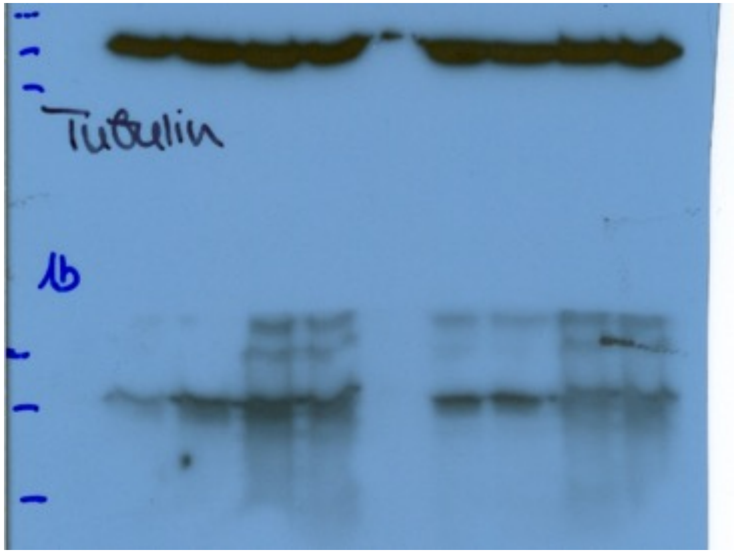

Anti-Tubulin

Anti-Bcl2

1 2 3 4 5 6

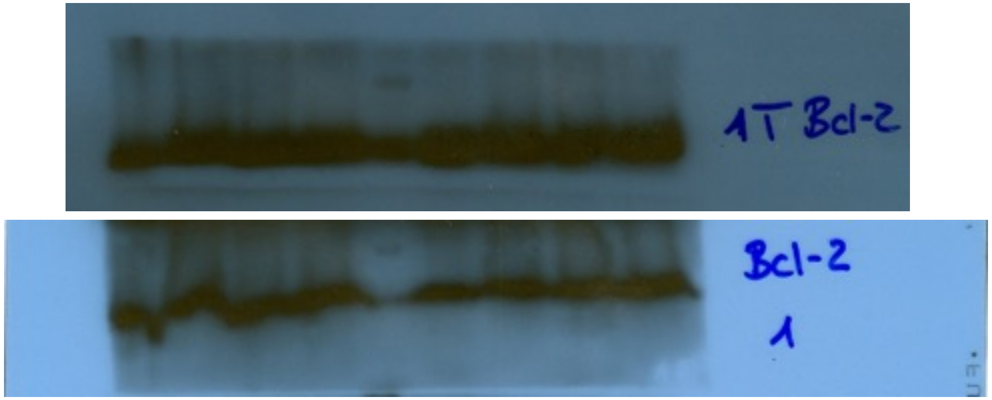

7 8

miR-148a
Bax

Publication

anti-Bax (miR-148/K270+K410)

1 K270: Scr
2 K270: miR-148a Mimic
3 K270: Scr
4 K270: miR-148a Inhibitor
5 K410: Scr
6 K410: miR-148a Mimic
7 K410: Scr
8 K410: miR-148a Inhibitor

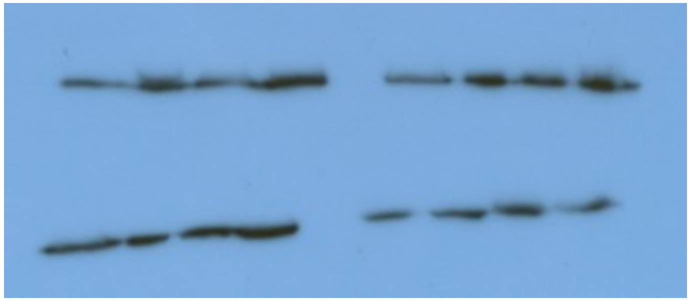

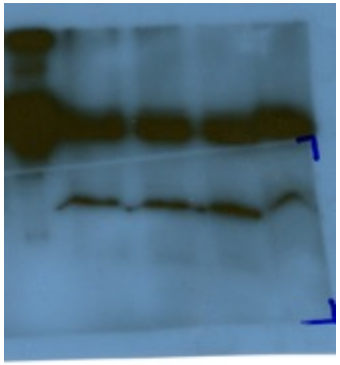

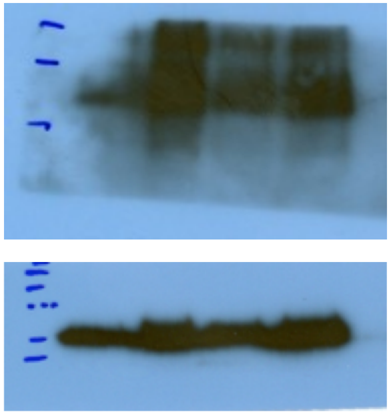

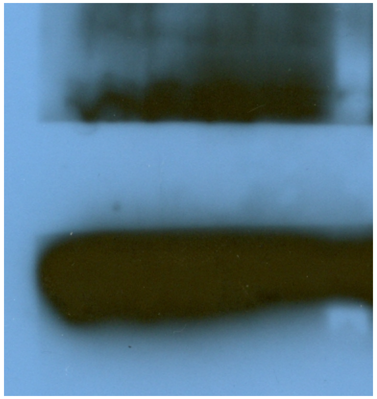

Brightness: +30%
Contrast: +20%

Second and third experiment

Anti-Bax (miR-148a/K270)

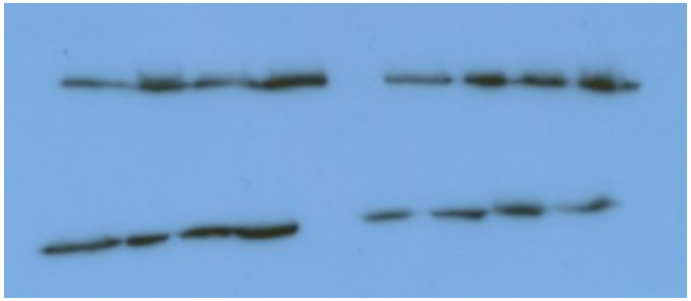

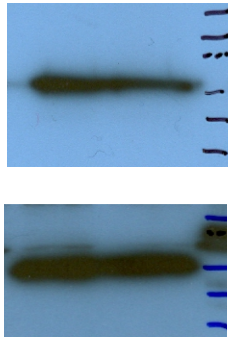

1 K270: Scr
2 K270: miR-148a Inhibitor
3 K270: Scr
4 K270: miR-148a Inhibitor
5 K270: Scr
6 K270: miR-148a Mimic
7 K270: Scr
8 K270: miR-148a Mimic

Second and third experiment

Anti-Bax (miR-148a/K410)

1 K410: Scr
2 K410: miR-148a Mimic
3 K410: Scr
4 K410: miR-148a Inhibitor
5 K410: Scr
6 K410: miR-148a Inhibitor
7 K410: Scr
8 K410: miR-148a Mimic

1 2 3 4

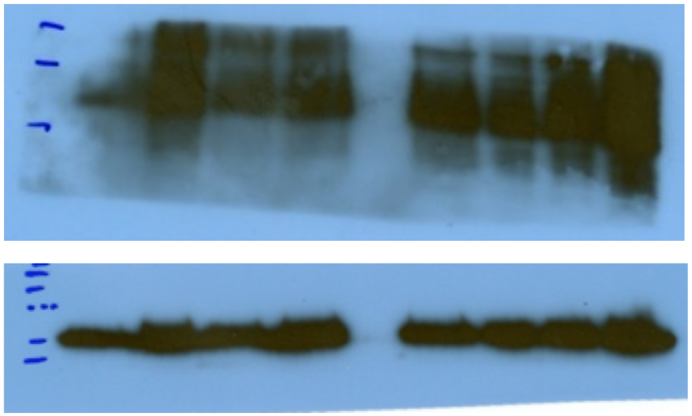

Anti-Bax

Anti-Tubulin

5 6 7 8

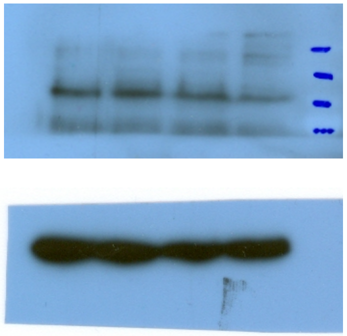

Anti-Bax

Anti-Tubulin
